# ACE2 receptor polymorphism in humans and animals increases the risk of the emergence of SARS-CoV-2 variants during repeated intra- and inter-species host-switching of the virus

**DOI:** 10.3389/fmicb.2023.1199561

**Published:** 2023-07-13

**Authors:** Christian A. Devaux, Jacques Fantini

**Affiliations:** ^1^Laboratory Microbes Evolution Phylogeny and Infection (MEPHI), Aix-Marseille Université, IRD, APHM, MEPHI, IHU–Méditerranée Infection, Marseille, France; ^2^Centre National de la Recherche Scientifique (CNRS-SNC5039), Marseille, France; ^3^INSERM UMR_S1072, Marseille, France, Aix-Marseille Université, Marseille, France

**Keywords:** SARS-CoV-2, spike, ACE2, zoonoses, genetic drift, selective pressure

## Abstract

Like other coronaviruses, SARS-CoV-2 has ability to spread through human-to-human transmission and to circulate from humans to animals and from animals to humans. A high frequency of SARS-CoV-2 mutations has been observed in the viruses isolated from both humans and animals, suggesting a genetic fitness under positive selection in both ecological niches. The most documented positive selection force driving SARS-CoV-2 mutations is the host-specific immune response. However, after electrostatic interactions with lipid rafts, the first contact between the virus and host proteins is the viral spike-cellular receptor binding. Therefore, it is likely that the first level of selection pressure impacting viral fitness relates to the virus’s affinity for its receptor, the angiotensin I converting enzyme 2 (ACE2). Although sufficiently conserved in a huge number of species to support binding of the viral spike with enough affinity to initiate fusion, ACE2 is highly polymorphic both among species and within a species. Here, we provide evidence suggesting that when the viral spike-ACE2 receptor interaction is not optimal, due to host-switching, mutations can be selected to improve the affinity of the spike for the ACE2 expressed by the new host. Notably, SARS-CoV-2 is mutation-prone in the spike receptor binding domain (RBD), allowing a better fit for ACE2 orthologs in animals. It is possibly that this may also be true for rare human alleles of ACE2 when the virus is spreading to billions of people. In this study, we present evidence that human subjects expressing the rare E_329_G allele of ACE2 with higher allele frequencies in European populations exhibit a improved affinity for the SARS-CoV-2 spike N_501_Y variant of the virus. This may suggest that this viral N_501_Y variant emerged in the human population after SARS-CoV-2 had infected a human carrying the rare E_329_G allele of ACE2. In addition, this viral evolution could impact viral replication as well as the ability of the adaptive humoral response to control infection with RBD-specific neutralizing antibodies. In a shifting landscape, this ACE2-driven genetic drift of SARS-CoV-2 which we have named the ‘boomerang effect’, could complicate the challenge of preventing COVID with a SARS-CoV-2 spike-derived vaccine.

## 1. Introduction

Since the first described human infection with severe acute respiratory syndrome coronavirus type 2 (SARS-CoV-2; a Betacoronavirus lineage 2b/Sarbecovirus) which emerged in China in 2019 (Zhu et al., 2020; Frutos et al., 2020a; Zhou P. et al., 2020), genomic surveillance has revealed rapid expansion of novel SARS-CoV-2 variants over time (Alkhatib et al., 2021; Bano et al., 2022; Colson et al., 2022a). One of the earliest SARS-CoV-2 variants detected through genomic epidemiology was the D_614_G which rapidly became dominant around the world (Korber et al., 2020; Plante et al., 2020). This immediately caught the attention of and raised fear along the international medical community which was actively working to develop an anti-COVID-19 vaccine. The fact that sera from convalescent individuals showed effective cross-neutralization of both the wild type SARS-CoV-2 and D_614_G variants (Garcia-Beltran et al., 2021; Legros et al., 2021), had allayed concerns about the risk of escape from immunity acquired after infection or vaccination. However, the identification of this first variant was only the harbinger of a succession of new lineages. The medical community could do nothing but observe the rapid expansion of a new lineage harboring three amino acid deletions and seven missense mutations in the spike, including D_614_G and N_501_Y in the ACE2 receptor-binding domain (RBD). This variant of concern (VOC), termed B.1.1.7 (also known as VOC-202012/01 or 501Y.V1 or Alpha), which first emerged in the United Kingdom, was reported to be more infectious than the D_614_G variant (Tang et al., 2020; Galloway et al., 2021). Fortunately, sera from convalescent patients continued to cross-neutralize B.1.1.7 variants, with only slightly decreased neutralizing potency (Bates et al., 2021; Muik et al., 2021; Rees-Spear et al., 2021; Weissman et al., 2021). Notably, this lineage harbors a mutation (N_501_Y) in its Spike (S) RBD that enhances binding to ACE2 (Barton et al., 2021). This result should have raised questions about the “passive” or “active” role of ACE2 in the emergence of new SARS-CoV-2 variants, since ACE2 alleles in humans are known to harbor amino acid substitutions in the ACE2 region acting as target for the SARS-CoV-2 S protein RBD. Over time, there was reports of numerous variants such as the B.1.429, containing four missense mutations in the spike, one being a single L_452_R RBD mutation, and the B.1.351 (Beta) lineage (also known as 501Y.V2) which first emerged in South Africa bearing three mutations, K_417_N, E_484_K, and N_501_Y in the RBD, and several mutations outside the RBD (Hoffmann et al., 2021; Tegally et al., 2021). This 501Y.V2 variant was a source of concern for the medical community, since it was considered to be less sensitive to immune response cross-neutralization (Hu et al., 2021; Shen et al., 2021; Thye et al., 2021; Parums, 2023; Wang et al., 2023). Notably, the D_215_G mutations found in the Beta lineage were found to induce partial resistance to neutralization (McCallum et al., 2021). The B.1.617 (Delta) then emerged in India and was found to partly evade neutralization (Samarasekera, 2021; Yue et al., 2021). Evasion to neutralization was also observed with the SARS-CoV-2 omicron (B1.1.529) variant (Sheward et al., 2022) as well as the XBB and XBB.1 subvariants of SARS-CoV-2 Omicron BA.2 and the BQ.1 and BQ.1.1 subvariants of BA.5 (Wang et al., 2023; Zhu et al., 2023). The Omicron XBB.1.5 (“Kraken”) subvariant is a sublineage of the XBB variant, a recombinant of two BA.2 sublineages, with the F_486_P mutation in the S protein that increases infectivity due to increased binding affinity to ACE2 (Parums, 2023). Interestingly, we found that most variants exhibit concomitant mutations in the RBD and in the N-terminal domain (NTD) sequences, both domains acting synergistically to ensure optimal virus adhesion (Fantini et al., 2021a). Some mutations affect the affinity of the spike protein for ACE2, while other mutations increase the electropositive surface of the S protein, with drastic effects on the kinetics of virus adhesion to lipid raft gangliosides.

These examples illustrate the genetic drift of SARS-CoV-2 and the inability of the scientific and medical communities to understand and anticipate the lineage replacement during the ongoing SARS-CoV-2 pandemic. Notably, at the beginning of the pandemic, early claims considered that the genetic diversity of SARS-CoV-2 should be extremely low (Dearlove et al., 2020; Rauch et al., 2022).When we become aware of the genomic variations of SARS-CoV-2 during the pandemic, the first question that arose was whether this genetic drift of SARS-CoV-2 was stochastic, whether it highlights the positive selection of variant within the SARS-CoV-2 quasi species, or both. If there was a positive selection process four questions were raised. First, is it only associated with the host-specific immune response?; Second, could it depend on the virus affinity for the ACE2 receptor?; Third, are interspecies transmission of SARS-CoV-2 determinant for virus evolution?; And finally, does the minor human ACE2 allele (and large size of the infected human population) contribute towards the emergence of SARS-CoV-2 variants? Answers to these questions enabled us to propose the ACE2-driven “boomerang effect” model, which hypothesizes that the SARS-CoV-2 replicates in individual A bearing the (a) ACE2 receptor, infects individual B bearing the (b) ACE2 receptor, mutates to adapt to the (b) ACE2 receptor of individual B, and returns to the population of A individuals with different properties.

## 2. Intra- and inter-species spread of putative VOCs: the ‘boomerang effect’

SARS-CoV-2 is evolving through a quasispecies mechanism which was reported earlier for SARS-CoV-1 and the other coronaviruses (Xu et al., 2004; Karamitros et al., 2020). Viral quasispecies are deeply influenced by the rate of nucleotide (nt) misincorporation per nt copied and their adaptability for the invasion of tissues and organs (Domingo et al., 2012; Jary et al., 2020). A significant characteristic of the quasispecies evolutionary process is the generation of post-infection mutations under positive selective pressure (host-driven viral evolution). Although intra-host analysis of SARS-CoV-2 evolution has revealed the existence of one master mutant and numerous minor mutants in quasispecies, minor mutants may obtain a fitness advantage and become the master mutants under high selective pressure (Sun et al., 2021). This provide a rational for a minor mutant selection when changing host. The RNA-dependent RNA polymerase (*RdRp*) gene of coronaviruses is known to be error-prone, thereby leading to frequent mutation and recombination (Chen, 2020). The mutation rate of SARS-CoV-2 substitutions was originally estimated to be 4.83 ×10^−4^/site/year (Mercatelli and Giorgi, 2020). It was reported that SARS-CoV-2 with a genomic mutation C14408U causing a P_314_L substitution in the *RdRp (nsp12)* increases errors by a factor of three (Pachetti et al., 2020). It was also found that the substitution P_203_L in the exonuclease proofreading subunit (nsp14) increases errors by a factor of two (Takada et al., 2023). Notably one recent report established that the fidelity of nsp12, along with its co-factors nsp7 and nsp8 and in the absence of nsp14 is 10^−1^–10^−3^ to be compared to a fidelity of 10^−6^–10^−7^ for other coronaviruses. This is likely to be due to critical mutations in nsp12 and nsp14 (Yin et al., 2023). It suggests that SARS-CoV-2 has a fairly high ability for fitness, which is advantageous for new host viral adaptation if the virus should interact with a cellular receptor exhibiting some degree of polymorphism.

**Figure 1:**
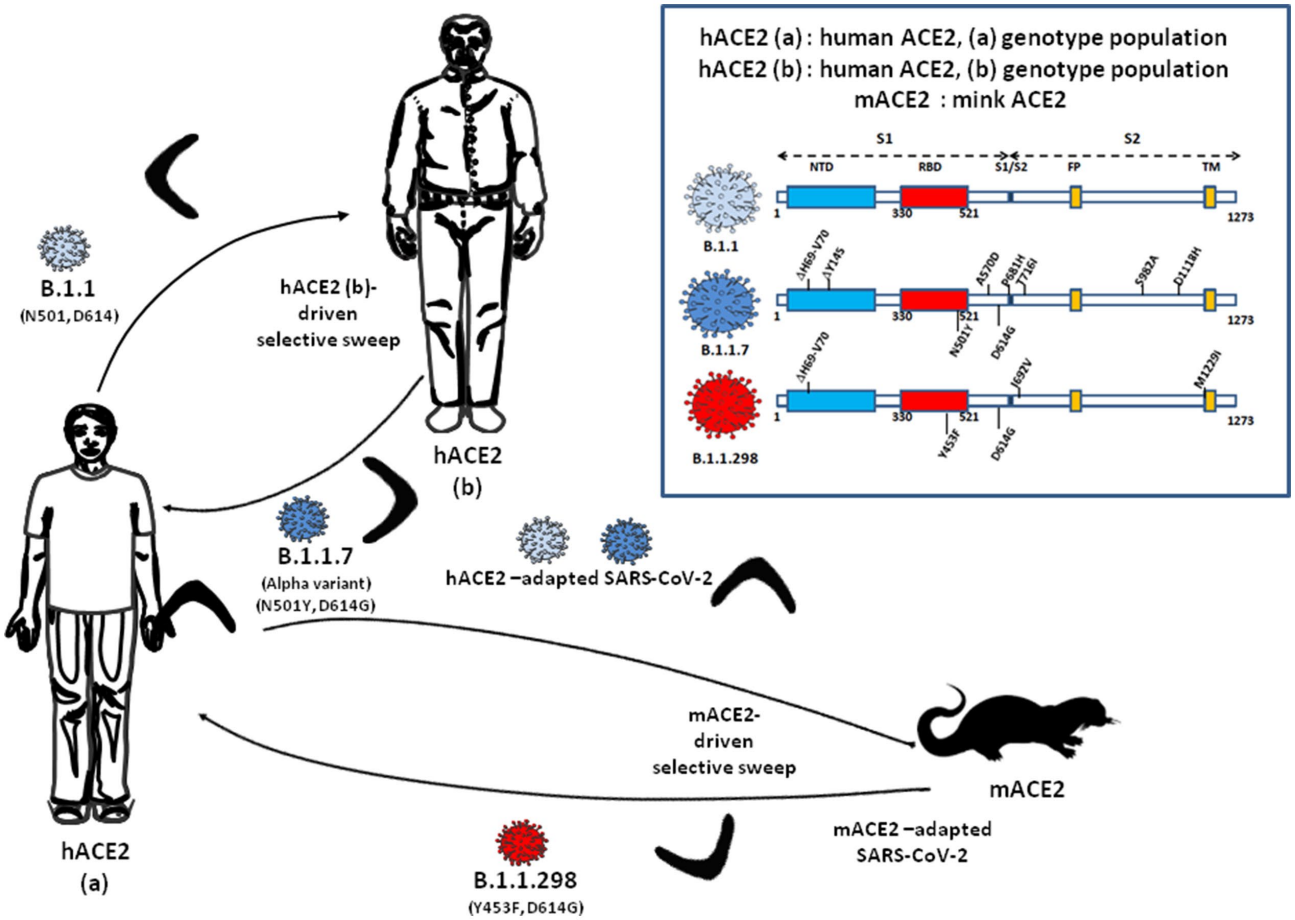
Illustration of the ACE2-driven “Boomerang effect” that positively selects SARS-CoV-2 variants for their binding affinity to the host ACE2 during intra- (human to human) and inter-species (human-to-mink and mink-to-human) transmission of the virus. Box (upper right): schematic representation of the SARS-CoV-2 spike (S) protein from B1.1 (1,273 amino acids), B1.1.7 and B1.1.298 lineages. The Spike (S) protein is comprised of an N-terminal subunit (S1) that mediates the receptor binding and a C-terminal subunit (S2) responsible for virus-cell membrane fusion. NTD: N-terminal domain; RBD, receptor-binding domain; FP, fusion peptide; TM, single-span transmembrane domain.

Besides the inter-human transmission of SARS-CoV-2 among hosts who are expected to share relatively conserved ACE2 viral receptors, the report of back-and-forth SARS-CoV-2 transmission between humans and animals with spillback in humans of a variant known as mink cluster 5 or B.1.1.298, harboring a two-amino acid deletion and four missense mutations including an Y_453_F substitution in RBD, was more surprising (Frutos and Devaux, 2020; Hammer et al., 2021; Oude Munnink et al., 2021). Novel variants were described containing an E_484_K mutation in the RBD, which was previously identified through *in vitro* selection experiments to escape from monoclonal antibody neutralization (Baum et al., 2020; Greaney et al., 2021a,b) arising from the B.1.1.28 lineage. These variants, were termed P1 (Gamma) and P2. The P.2 variant harbored three spike missense mutations while P.1, which first emerged in Brazil, harbored 12 spike missense mutations including the K_417_T and N_501_Y substitutions in RBD. These strains spread rapidly even among individuals who had been previously infected with another lineage of SARS-CoV-2 (Paiva et al., 2020; Brouqui et al., 2021; Goes et al., 2021; Menezes et al., 2022). The detailed affinity analysis of five common RBD mutations (K_417_N, K_417_T, N_501_Y, E_484_K, and S_477_N) and two common mutations (S_19_P and K_26_R) on the viral spike/ACE2 interaction, indicated that apart from K_417_N/T which decreased the affinity and facilitated immune escape, the other mutations increased the affinity of the RBD for ACE2 (Barton et al., 2021; Bates et al., 2021; Hoffmann et al., 2021; Sheward et al., 2022; Yue et al., 2023). The K_417_N/T substitution was also detected in the B.1.617.2 (Delta) VOC and its sublineages (AY.1 and AY.2). The fact that a subset of the variants including B.1.1.7 (Alpha) and B.1.617.2 (Delta), increases viral transmission, while others, such as B.1.351 (Beta), P1 (Gamma), and B.1.526 (Lota), escape humoral immunity, raises the question of a possible ‘boomerang effect’ of repeated intra- and/or inter-species transmission of SARS-CoV-2, increasing the risk of reintroducing VOCs which were less susceptible to antibody neutralization after ACE2-driven selective sweep, into human population (Figure 1). Therefore, contrary to the usual hypothesis of the strong selection of variants by the host immune system, is there enough experimental evidence to support the hypothesis that at least some SARS-CoV-2 variants would have been generated following a process which would align with the ACE2-driven “boomerang effect” model?

## 3. Compatibility of the SARS-CoV-2 omicron variant with highly divergent ACE2 orthologs

Question regarding the hypothesis of the ACE2-driven “boomerang effect” model became more pressing with the observation of the outbreak of the SARS-CoV-2 VOC Omicron B.1.1.529 lineage (Pango or BA.1). The spike protein of this lineage contained 45 point mutations compared with the B1.1 lineage, including seven changes in the NTD, a scale of mutations never previously observed with the other SARS-CoV-2 lineages. These seven mutations (namely K_417_N, G_446_S, E_484_A, Q_493_R, G_496_S, Q_498_R, and N_501_Y) were located at the interface of ACE2 and the spike protein RBD and many of them (such as N_501_Y) correspond to those needed for adaptation to the murine ACE2 (Wei et al., 2021). Compared with the B.1.617.2 (Delta) variant, the RBD of B.1.1.529/Omicron has an increased electrostatic surface potential, but a decreased affinity for the ACE2 receptor and its NTD has both a decreased surface potential and a lower affinity for lipid rafts (Fantini et al., 2022a). Consequently, the Omicron variant was predicted to be less fusogenic and thus less pathogenic than the Delta variant, due to a structural reorganization of the S1-S2 cleavage site. The spike from Omicron is believed to have been subjected to a strong positive selection in a non human host species (Wei et al., 2021). However, the B1.1.529 isolates induced only mild loss body weight and a lower viral burden in the respiratory tracts compared to B.1.351 (Halfmann et al., 2022), suggesting a co-evolution of Omicron and its possible murine host. Notably, Omicron show the P_323_L mutation in nsp12 and I_42_V in nsp14 which possibly contribute to its high mutation rate (Jung et al., 2022). Omicron is a promiscuous virus that has spread to many species and which has shown high propensity to escape antibody neutralization (Mallapaty, 2022; Planas et al., 2022). This was further confirmed with Omicron subvariants B.1.1.529.2/BA.2, BA.2.12.1, B.1.1.529.3/BA.3, B.1.1.529.4/BA.4 and B.1.1.529.5/BA.5 (Cao et al., 2022; Hachmann et al., 2022).

**Figure 2:**
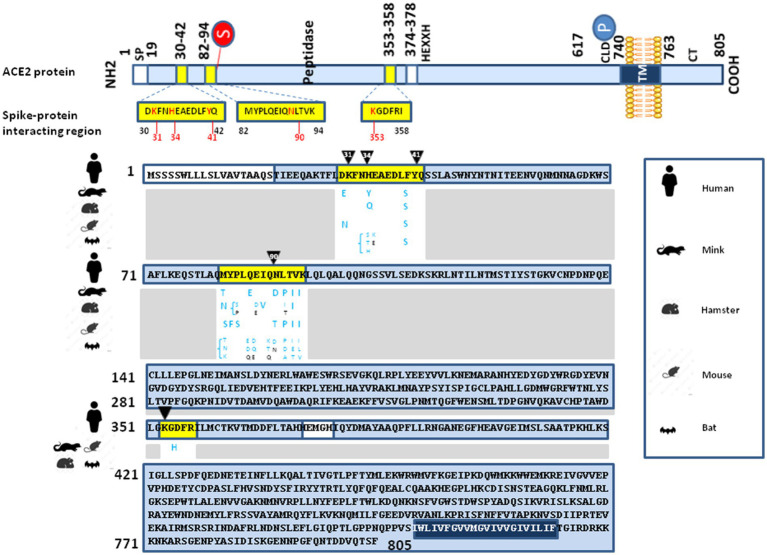
Illustration of interspecies ACE2 polymorphism. (upper panel) Schematic representation of the cell surface human ACE2 molecule (a type I transmembrane glycoprotein of ∼100 kDa composed of 805 amino acids) and its major domains (the N-terminal signal peptide region; the peptidase domain, amino acids 19–615, with its zinc binding metalloprotease motif HEXXH, amino acids 374–378; the C-terminal collectrin-like domain, amino acids 616–740; and, the hydrophobic transmembrane hydrophobic helix region of 22 amino acids followed by an intracellular cytoplasmic tail of 43 amino acids). The main spike protein interacting region are depicted in yellow boxes. The amino acid positions are in indicated black. Some of the amino acids considered to be important for viral tropism are marked in red. (lower panel) Comparison between the *Homo sapiens* ACE2 protein sequence (Genbank BAB40370.1) and the sequences from minks, hamsters, mice and bats using Clustal Omega multiple sequence alignment, as previously described (Devaux et al., 2021a,b). All sequences obtained from the NCBI reference sequence database were previously reported in our previous publications (Devaux et al., 2021a,b; Frutos et al., 2022; Fantini et al., 2022b). The figure only shows the polymorphism within the main spike protein interacting region (blue letters). In the white boxes, the black letters (next to the blue letters) indicate that in this species an amino acid identical to that found in the sequence of human ACE2 can also be found although there is usually a polymorphism with respect to human ACE2 at this position in this species. Recently additional bat ACE2 variants have been reported (Li et al., 2023), and they are not all illustrated in this figure.

It was previously determined that the main spike-interacting region on the surface of ACE2 comprised amino acids 30 to 42, 82 to 94 and 353 to 358) (Lan et al., 2020; Shang et al., 2020; Yan et al., 2020; Suryamohan et al., 2021) As can be seen in Figure 2, when comparing the spike-interacting region of the human ACE2 and murine ACE2 they show 69% of identity (10 positions with different amino acids on 32 amino acids). An ancestral human lineage of Omicron may very well have infected mice before evolving specifically in this rodent species and infecting back humans as the Omicron lineage (Fantini et al., 2022a). Notably, the L_452_R mutation present in the B.1.617.1 (Kappa), B.1.617.2 (Delta), and B.1.427 (Epsilon) variants was associated with a modest increase in infectivity as measured after incubation with soluble murine ACE2 (Liu et al., 2021). This strongly suggests a process of ACE2-driven selective sweep. In genetics, a selective sweep is the process by which a new beneficial mutation increases its frequency and becomes fixed in the (viral) population leading to the decrease or elimination of genetic variation near the mutation. The lineage replacement observed with SARS-CoV-2 over the pandemic supports the hypothesis that SARS-CoV-2 evolved to fit the constraints of interacting with polymorphic ACE2 receptors and that when a mutation intended to improve this interaction in an individual or in a species is inconsequential or remains advantageous when changing individuals or species, it remains fixed. This “boomerang model” considers that viral variants rapidly arise during the *in vivo* passage of the virus to adapt its spike to the positive selection imposed by the ACE2 receptor of the host. Repeated intra- and inter-species transmission of SARS-CoV-2 present the potential for acceleration of genetic drift and a possible source of the emergence of novel strains. This was demonstrated by reverse zoonosis of SARS-CoV-2 from humans to minks, followed by selection in minks and zoonotic transmission back to humans (Hammer et al., 2021; Oude Munnink et al., 2021). The “boomerang effect” occurs when the variant reinfects the species of the first individual infected with a beneficial mutation that can be fixed.

## 4. Lessons drawn from ACE2-tropic Sarbecoviruses

Since the beginning of the COVID-19 pandemic, one major issue has been the origin of the virus and how it spread into human populations. Although evidence of the human-to-human transmission of SARS-CoV-2 was rapidly reported (Huang et al., 2020; Kucharski et al., 2020), explanations for the magnitude of the pandemic and the origin of the virus remains debated. However, most data supports the fact that SARS-CoV-2 is a naturally occurring virus circulating in the wild (a multi-host virus) which binds to an ubiquitous cellular receptor and came into contact with humans under a stochastic model (Frutos et al., 2021a). Previously, the closest sequences to SARS-CoV-2 characterized in wild animals were found in bats. The first one to have been identified was the RaTG13 sequence (96% of sequence identity with SARS-CoV-2), which was obtained from a *Rhinolophus affinis* bat (Zhou P. et al., 2020). Another BatCoV sequence RacCS203 (sharing 95.86% similarity with SARS-CoV-2), was found in a *Rhinolophus acuminatus* bat (Wacharapluesadee et al., 2021). The RmYN02 batCoV sequence (sharing 93.3% similarity with SARS-CoV-2), was identified in a *Rhinolophus malayanus* bat (Zhou H. et al., 2020). Two other SARS-CoV-2 related sequences, RshSTT182 and RshSTT200 (sharing 92.6% overall similarity with SARS-CoV-2), were described in *Rhinolophus shameli* bats (Delaune et al., 2021). Although bats are very often carriers of coronaviruses (Ge et al., 2013; Hu et al., 2015; Forni et al., 2017; Afelt et al., 2018; Frutos et al., 2021b), some of which are very similar to SARS-CoV-2, there is no indication that either a bat Sarbecovirus or a pangolin Sarbecovirus was the cause of pandemic SARS-CoV-2 in humans (Frutos et al., 2020b). Indeed, these viruses could have evolved in parallel within both species.

Yet, as shown in Figure 2, in the main spike-interacting region (amino acids 30–42, 82–94 and 353–358) there is a high degree of polymorphism between the bat ACE2 and ACE2 from other species. In addition, there is also a significant polymorphism among ACE2 from different bat species. Seven polymorphic ACE2 variants were recently reported in *Rhinolophus affinis* (Ra) bats, showing varying susceptibility to the entry of RaTG13 spike pseudovirions (Li et al., 2023). These authors reported that single D_501_N and H_505_Y substitutions in the RaTG13 spike protein significantly enhance infectivity and minimize the difference in susceptibility among different RaACE2 variants, while an N_501_D substitution in the SARS-CoV-2 S protein leads to a reduction in infectivity in several RaACE2 variants. Bats are the second largest order of mammals after rodents, with over 1,400 species and certain combinations of amino acids in RaACE2 may have favored the selection of viruses that could circulate more easily in inter-species and outside the bat order. Notably, while Y_505_ is present in the Alpha, Beta, Gamma, and Delta variants, H_505_ becomes dominant in most Omicron variants.

There is still no information on where and when the first contact between a SARS-CoV-2 ancestor and humans occurred. These events are complex to determine because the recognition of a novel disease does not start with a few cases (latency phase) but when an epidemic threshold or Critical Community Size (CCS) is reached. The search for the first contact between the ancestor of SARS-CoV-2 with a human would require a retrospective investigation with little chance of success. In addition, it should be borne in mind that the four genera of CoVs (α and β known to infect mammals, and γ and δ known to infect both mammals and birds) have been predicted to have diverged millions of years ago (Wertheim et al., 2013), and that the circulation of these viruses in different animal hosts has resulted in a myriad of recombination events (Simas et al., 2015; Corman et al., 2018; Zhou et al., 2018; Colson et al., 2022b; Focosi et al., 2023), shedding light on the probable dynamic of evolution that may also have applied to the SARS-CoV-2 ancestor.

## 5. The spread of SARS-CoV-2 relies on ACE2 receptor recognition

One conceptual difficulty to understanding the process of inter-species viral circulation arises from the definition of “species barrier.” The concept of “species barrier” refers to the idea that a species is an isolated entity within an ecosystem and that the transmission of viruses from one species to another requires crossing a barrier between them the biological nature of which is elusive. Following a shift in the original “spillover” definition proposed to describe the risk of epizootics in wildlife from livestock (Daszak et al., 2000), it was suggested that an animal virus reaches a high prevalence in a “reservoir” and then spills over into another host (such as humans), a process routinely referred to as “pathogen spillover” (Power and Mitchell, 2004). Later, the concept of spillover was further distorted to the point of being used as a synonym for contamination. However, looking at zoonosis through this prism is likely to be far from what can be seen by observing the ecosystem reality as defined by the “One Health” approach (Cardiff et al., 2008; Adisasmito et al., 2022; Keusch et al., 2022). The spillover model remains an anthropocentric concept that differentiates human targets on one side from animal species targets on the other. However, this does not make sense. A virus does not distinguish between species before spreading, it infects target species simply through its ability to recognize a receptor (here, ACE2) on a susceptible cell and then complete its cycle of replication if it can evade the host immune defenses (Frutos et al., 2021a). There is now a great deal of evidence in the literature of SARS-CoV-2 transmission from humans to a huge number of species (Conceicao et al., 2020; Shi et al., 2020; McAloose et al., 2021; Hale et al., 2022). According to the Vetmeduni/Complexity Science Hub, Vienna^1^ which compiled all the SARS-CoV-2 infection events in animals from January 2020 to December 2022, 31 animal species were found infected by one or more SARS-CoV-2 lineages (616 animal outbreaks of SARS-CoV-2 in 39 countries) (Supplementary Figure S1).

In addition to the immune responses of these species (and particularly the humoral/adaptive neutralizing antibody immune response), what became important in the context of SARS-CoV-2 inter-host circulation was the structural nature of the ACE2 viral receptor, which primarily plays a critical role in host homeostasis as well as acting as receptor for SARS-CoV-2 (Donoghue et al., 2000; Turner and Hooper, 2002; Towler et al., 2004; Devaux et al., 2020; Devaux and Camoin-Jau, 2022). This is why many teams have made efforts to characterize the polymorphism of ACE2 in different species (Damas et al., 2020; Luan et al., 2020; Qiu et al., 2020; Ren et al., 2021; Devaux et al., 2021a). ACE2 may have enough affinity for the SARS-CoV-2 spike to allow for binding and infection, but may also be a force of positive selection likely to promote mutations enabling greater affinity of the spike for the ACE2 of this host species and/or providing a kinetic advantage. During work carried out under the “One Health” approach to monitoring animals (especially in mass rearing facilities and companion animals), searching for possible SARS-CoV-2 infections, massive SARS-CoV-2 infections were discovered in mink farm animals resulting from the transfer of the virus from humans to animals (Oude Munnink et al., 2021). These mink farm epidemics led governments to order the culling of millions of animals due to the global fear that more pathogenic variants could emerge through *in vivo* passage in animals prior to re-infecting humans, a phenomenon referred to as the “boomerang effect.”

## 6. Risks associated with ACE2-driven VOCs selection followed by the “boomerang effect”

Eighteen years before the emergence of SARS-CoV-2, an outbreak of a related ACE2-tropic Sarbecovirus, SARS-CoV-1 (Li et al., 2003) occurred in the winter of 2002–2003, resulting in the death of approximately 800 people (Drosten et al., 2003; Ksiazek et al., 2003). Early cases of SARS-CoV-1 infection were found in Asian animal traders and restaurant workers handling wild mammals such as palm civets and raccoon dogs and nucleotide sequence variation in the S gene of animal and human SARS-CoV-1 were reported (Guan et al., 2003). Although highly conserved (mammal and human SARS-CoV-1 revealed 99.8% genomic sequence identity), the sequences of the SARS-CoV-1 S gene varies with some mutations that seemed critical for the transition from animal-to-human transmission to human-to-human transmission, particularly in the region predicted to constitute the RBD, including the N_479_ residue with K or R substitutions (Guan et al., 2003; Song et al., 2005). Years before the SARS-CoV-2 pandemic, this observation suggested that the RBD is under positive selection of the host species ACE2 receptor and that some substitutions are compatible with or favor transmission to a new host species. ACE2 from humans, mice and rats give the SARS-CoV-1 the ability to replicate in these species, however murine ACE2 less efficiently bound the S1 domain of SARS-CoV-1 and supported less efficient S protein-mediated infection, while rat ACE2 was even much less efficient than the murine ACE2 (Li et al., 2004; Subbarao et al., 2004; Wentworth et al., 2004). Li and colleagues (Li et al., 2005) compared the S protein of SARS-CoV-1 isolated during both the 2002–2003 outbreak and the much less severe 2003–2004 outbreak to the SARS-CoV spike from palm civets. They found that all three S protein can bind to and utilize palm-civet ACE2 efficiently, but the latter two S protein utilized human ACE2 markedly less efficiently than did the S protein obtained during the earlier human outbreak. The different binding capacities were associated with substitutions in the RBD residues 479 and 487. Although the emergence of SARS-CoV-1 remained limited in number of cases, it already demonstrated the major impact of positive selection by ACE2 in the inter-species viral circulation. In addition, this showed that SARS-CoV-1, a virus closely related to SARS-CoV-2, already had the ability to circulate between mice and humans much as the SARS-CoV-2 Omicron sub-variants do today.

Should we fear the “boomerang effect” and take measures to anticipate its harmful consequences? Most frequently the risk of harmful consequences in the event of the reintroduction of a variant virus after its adaptation to the ACE2 from a different species is low because the mutation or mutations are very likely to have “host-specific signatures.” According to the quasispecies evolutionary model, these mutations do not predate the new host infection and can be considered specific to the host and advantageous for the virus mainly in this host species (Woo et al., 2012; Frutos and Devaux, 2020). Therefore, the SARS-CoV-2 variants found in minks in Denmark were very likely to be the result of “mink signatures.” Variants such as the mink-selected SARS-CoV-2 Y_453_F and D_614_G or H_69_del/V_70_del, Y_453_F, I_692_V and M_1229_I were identified in humans after spreading through densely caged minks. Maintenance of these mutations in humans suggests that they are either neutral or also advantageous in the human context.

## 7. Finding the best fit for the mink, hamster and deerACE2

**Figure 3:**
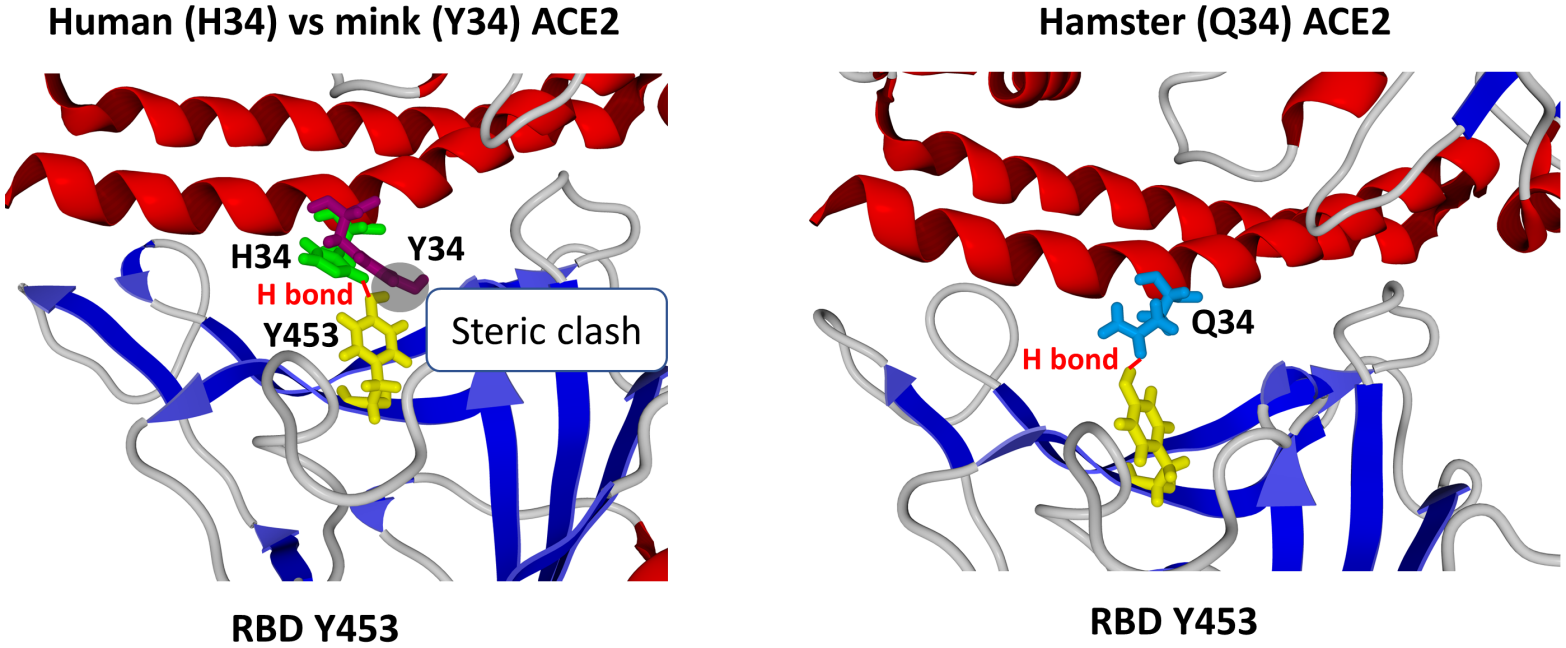
Impact of the type of the amino acid used at position 34 of animal ACE2 on the interaction with the SARS-CoV-2 spike protein. Left panel: superposition of human ACE2 (H_34_) and EU mink ACE2 (Y_34_). H_34_ interacts with the spike protein residue Y_453_ through a H-bond. However, in the case of minks, the H_34_ is substituted by Y_34_, which is too large to accommodate the side chain of Y_453_. This steric clash is resolved by the selection of a mutant spike at position 453 (the Y_453_F substitution). Right panel: In the case of hamster ACE2, the H_34_ is substituted by Q_34_, which does not clash with the spike residue Y_453_ and allows the formation of a H-bond. Secondary mutations in the spike protein (not shown) further stabilize this H-bond, which may confer a selective advantage when the virus comes back to humans, as previously reported (Fantini et al., 2022b). RBD, receptor binding domain.

A critical interaction in the SARS-CoV-2 infection cycle is the binding of the homotrimeric complex of viral S proteins to the peptidase domain of ACE2 (Lan et al., 2020; Shang et al., 2020; Yan et al., 2020). This interaction is driven by two domains located in the S1 subunit of the molecule, namely the RBD and the N-terminal domain (NTD). The NTD displays a flat electropositive ganglioside binding site enabling the virus to interact with lipid rafts of the cell membrane (Fantini et al., 2021b). At the N terminus of the viral spike, Q_498_, W_500_, and N_501_ of the RBD form a network of H-bonds with Y_41_, Q_42_, K_353_, and R_357_ of the human ACE2. In the middle of the bridge, K_417_ and Y_453_ of the RBD interact with D_30_ and H_34_ of ACE2, respectively. Moreover, Q_474_ of the RBD is H-bonded to Q_24_ of ACE2, whereas F_486_ of the RBD interacts with M_82_ of ACE2 through van der Waals forces (Yan et al., 2020). The human ACE2 key residues include S_19_, Q_24_, T_27_, F_28_, D_30_, K_31_, H_34_, E_35_, E_37_, D_38_, Y_41_, Q_42_, L_45_, L_79_, M_82_, Y_83_, T_324_, Q_325_, G_326_, E_329_, N_330_, K_353_, G_354_, D_355_, R_357_, P_389_, and R_393_ (Suryamohan et al., 2021). The K_31_ and K_353_ residues in human ACE2 form hydrogen bonds with the main chain of N_501_ and Q_493_ in the RBD. Several ACE2 substitutions were reported to increase cell susceptibility to SARS-CoV-2 while others were predicted to be less sensitive to SARS-CoV-2 (Shukla et al., 2021; Suryamohan et al., 2021; Devaux and Camoin-Jau, 2022). Instead of the H_34_ amino acid found in the human ACE2, the mink ACE2 presents a Y_34_ amino acid. H_34_ is essential for interaction with the Y_453_ residue in the RBD of the SARS-CoV-2 spike protein. The Y_453_F substitution in SARS-CoV-2 spreading in minks is a consequence of mink ACE2-driven selective sweep which abolishes this conflict. We recently reported (Frutos et al., 2022) that the amino acid residue Y_453_ in the RBD of human strains of SARS-CoV-2 generated an optimal interaction with the viral cell receptor when faced with H_34_ in the human ACE2. The oxygen atom borne by the phenolic group of tyrosine was at 2.1 Å from one of the protonated atoms of nitrogen of the imidazolium group, consistent with the establishment of an H-bond. The mobility of the histidine ring was facilitated by the CH2 group of H_34_, allowing a favorable orientation of the H_34_ and Y_453_ side chains. Despite being similar to the human ACE2 3D structure, the mink ACE2 electrostatic charges were less electronegative than those of the human ACE2. When Y_453_ in the virus spike was facing a Y_34_ in the mink ACE2, a steric hindrance prevents the establishment of the stabilizing interaction between the RBD and ACE2. The oxygen atom of the phenolic group of Y_453_ scratches the aromatic ring of Y_34_ in mink ACE2, while the Y_453_F substitution observed in the B1.1.298 SARS-CoV-2 strains spreading in minks suppressed this steric hindrance and restored an optimal binding with the mink ACE2 with both aromatic rings adopting a perpendicular orientation characteristic of T-shaped CH-pi stacking. The aromatic side chains of Y_34_ and F_453_ were separated by 3 Å, a distance fully consistent with this type of interaction. During the reverse infection of humans by mink-adapted-SARS-CoV-2, a reverse adaptation is observed that did not affect the amino acid 453 of the S protein but results in the appearance of compensatory mutations in other domains of the protein having a potentiating effect on the dynamic of the virus-cell interaction. The lack of a reverse mutation can likely be explained by a reduced conflict between H_34_ and F_453_ than between Y_34_ and Y_453_ (Figure 3).

A quite similar scenario has been reported in connection with the discovery of SARS-CoV-2- infected hamsters in Hong Kong (Kok et al., 2022; Fantini et al., 2022b). A SARS-CoV-2 Delta variant circulating in hamster was able to re-infect humans and to undergo human to human transmission (Kok et al., 2022). Specific substitutions were described in this hamster-adapted Delta variant of SARS-CoV-2, including a mutation T_38_I located in ORF10 and three mutations that affect the S protein. Two of the mutations, L_18_F and H_49_Y, were located in the N-terminal domain of the S protein. The D_427_G substitution was located inside the RBD but outside the region known to interact with the ACE2 receptor. The D_427_G mutation was found to be of particular importance for the virus binding to its receptor in both hamsters and humans (Fantini et al., 2022b). In the hamster ACE2 protein, H_34_ is replaced by Q_34_ which can still interact with Y_453_ through an H-bond. However, this generates a torsion in the protein structure pushing the amide group of Q_34_ in a direction opposite to that of H_34_. The D_427_G mutation annihilates this structural conflict. This mutation breaks the H-bond between D_427_ and G_413_. The α-helix is converted to a more flexible loop leading to a NH-π interaction between the residue N_422_ and the aromatic ring of Y_453_. Y_453_ is therefore attracted by the RBD, allowing the space required for the side chain of Q_34_ to adopt the initial orientation found with H_34_. This stabilizes the H-bond between Y_453_ and Q_34_ and reduces the distance between Q_34_ and D_427_ and G_427_, to 2.7 Å and 1.6 Å, respectively. These mutations that improve hamster ACE2 binding are apparently still advantageous for the virus in humans in whom the H-bond between Y_453_ and H_34_ remains optimized. The distance to Y_34_ is reduced from 3.5 Å to 2.7 Å and 2.2 Å for D_427_ and G_427_, respectively. The conformational change also has an impact upon the aromatic ring of F_486_ in the RBD, restoring the optimal energy of interaction (Fantini et al., 2022b) (Figure 3). Due to properties regarding its interactions with both hamster ACE2 and human ACE2, this SARS-CoV-2 Delta/Hong Kong variant could have been a variant with harmful consequences in the event of a “boomerang effect” leading to reinfection of a the human population. Fortunately, the surveillance and isolation measures applied by the local health authorities in Hong Kong prevented this virus from spreading among the human population.

**Figure 4:**
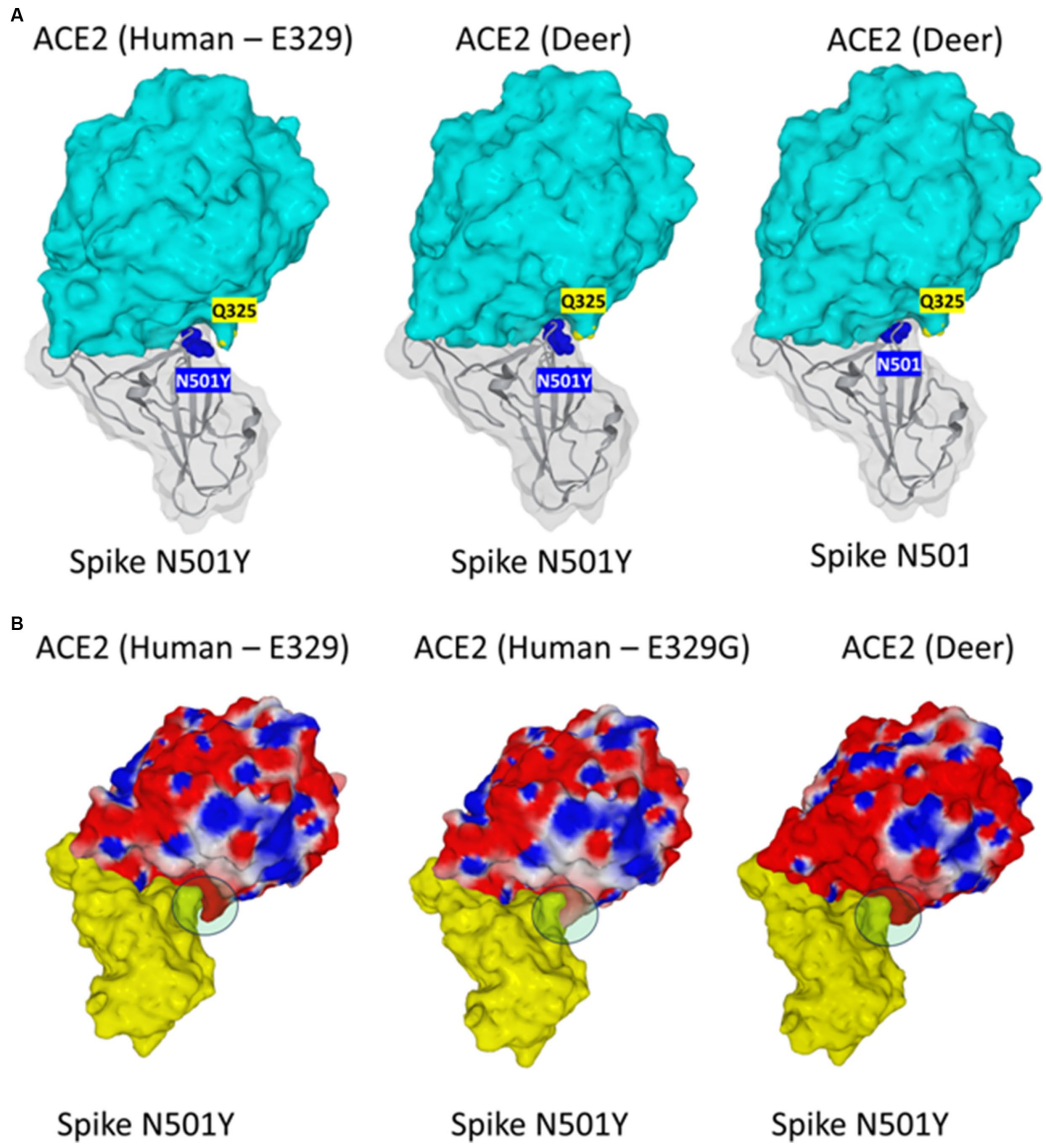
The figure illustrates the interaction of SARS-CoV-2 S protein with *Homo sapiens* ACE2 (GenBank: BAB40370.1) and deer (*Cervus Elaphus*) ACE2 (NCBI databank: XP_043752042.1). **(A)** The N_501_Y Spike has a better fit for deer ACE2 than for human (E_329_ isoform) ACE2, due to a better interaction between N_501_Y and Q_325_. The distance between N_501_Y and Q_325_ is shorter for deer ACE2 (middle panel) than for human ACE2 (left panel). Interestingly, the N_501_ Spike protein has a weaker affinity for deer ACE2 compared with N_501_Y. This is clearly visible in the right panel. N_501_ and N_501_Y are represented in blue atomic spheres, Q_325_ in yellow. The Spike protein is shown in gray ribbons with a transparent surface rendition in gray. ACE2 proteins are represented in a cyan surface rendition. **(B)** The affinity of deer ACE2 for the N_501_Y Spike is intermediate between human (E_329_ isoform) ACE2 and human (E_329_G). The region of interaction between N_501_Y and Q_325_ is indicated by a green disk. Note that the fit in this region is loose for human E329 (left panel), but in contrast, tight for the E_329_G (middle panel). In the case of deer ACE2 (right panel), the fit is also tight but the interaction area between N_501_Y and Q_325_ is smaller than for E_329_G. The Spike protein is represented with a yellow surface rendition. ACE2 proteins are represented in surface electropotential colors (blue, positive; red, negative; white, neutral). Note that the deer ACE2 is more electronegative than the human ACE2 proteins, suggesting a more rapid interaction with the Spike protein of SARS-CoV-2.

Human-to-deer transmission events have also been observed (Hale et al., 2022). Notably, it was reported that white-tailed deer (*Odocoileus virginianus*), the predominant cervids in North America, are highly susceptible to SARS-CoV-2 (D_614_G variant) infection and shed high viral titers in their tissues and secretions (Palmer et al., 2021; Kuchipudi et al., 2022). Immunoglobulin binding to SARS-CoV-2 were found in ~40% of wild deer sampled in various states in the US (Chandler et al., 2021; Palermo et al., 2022) and virus circulation was evidenced in different cervids including *Odocoileus virginianus*, *Odocoileus hemionus*, *Elaphurus davidianus*, and *Rangifer tarandus* (Martins et al., 2022). In Ontario (Canada), a SARS-CoV-2 was isolated in a sample of white-tailed deer and this virus was found to belong to a highly divergent lineage of SARS-CoV-2 (B.1.641 variant, with 76 mutations including 37 previously associated with non-human mammalian hosts), suggesting sustained evolution of SARS-CoV-2 in deer and deer-to-human transmission (Pickering et al., 2022). For the spike, the substitution reported are T_22_I, H_49_Y, T_95_I, V_143_-, Y_144_-, Y_145_D, S_247_G, F_486_L, N_501_T, Q_613_H, D_614_G, V_705_A, L_1265_I. With the same approach as before, we recently compared the deer ACE2 of *Cervus Elaphus* with the human ACE2 (unpublished data). We found many differences (142 mutated positions out of 805; a variability of 17.6%) with respect to the reference sequence chosen for the human ACE2 (Supplementary Figure S2). In the regions of ACE2 used for the attachment of the viral S protein to its receptor, several substitutions were found in the deer ACE2 including D_30_E (also found in mink ACE2), F_40_S (found in mink, hamster, mice, and bat ACE2), M_82_T (found in hamster and bat ACE2), Q_86_E (found in mink, mice, and bat ACE2), P_84_S (found in hamster and mice ACE2), K_31_N (found in mice ACE2) and V_93_L (apparently specific of deer ACE2). Despite this high polymorphism, the structural analysis of the interactions between the deer ACE2 and the S protein of SARS-CoV-2 shows that the deer ACE2 appears highly competent for SARS-CoV-2 interaction and there is a clear selective advantage for viruses that carry the N_501_Y substitution over those that have an N_501_. The distance between N_501_Y and Q_325_ was found to be shorter for deer ACE2 than for human ACE2 (Figure 4). When we analyzed the interaction between ACE2 and the viral S protein taking into account F_486_L and N_501_T substitutions, we observed that variants which would carry these substitutions have a better affinity for the deer ACE2 than for the human ACE2 but that these substitutions nevertheless remains less effective than the N_501_Y in favoring the S protein binding to deer’s ACE2 (data not shown). Notably, the substitutions F_486_L and N_501_T were recently found in SARS-CoV-2 isolated in two mink farms in late 2022 and early 2023 in Poland. The closest match was with lineage B1.1.307(GR/20B) but this variant had at least 40 nucleotide changes including several mutations in the spike gene leading to amino acid substitutions W_64_L, F_486_L, N_501_T, T_572_I and S_929_I and a deletion of four amino acids at positions 140–143 (Domańska-Blicharz et al., 2023). A previous publication already identified the mutations expected to be crucial for SARS-CoV-2 infection in minks as Y_453_F, F_486_L, N_501_T and D_614_G (Barua et al., 2022).

## 8. Human ACE2 polymorphism supports the hypothesis that the ACE2-driven “boomerang effect” could occur during human-to-human transmission of SARS-CoV-2

Long before the emergence of SARS-CoV-2, exploration of the *ACE2* genetic polymorphism in humans was conducted to define the single nucleotide polymorphisms (SNPs) associated with hypertension, coronary heart disease, and diabetes (e.g., rs2048683 and rs233575 were linked to moderate risks of hypertension, while rs4646188 and rs879922 were linked to high hypertension risks, and both rs2074192 and rs2106809 were associated with left ventricular hypertrophy in hypertensive patients) (Yang et al., 2015). As COVID-19 emerged, it was postulated that human susceptibility to SARS-CoV-2 infection could be affected by *ACE2* gene polymorphism (allele frequency heterogeneity) in different human subpopulations (Darbani, 2020; Devaux et al., 2020; Khayat et al., 2020). Dozens of human *ACE2* variants were identified which could impact ACE2 protein stability (e.g., K_26_R, G_211_R, and N_720_D variants) or internalization (e.g., L_351_V and P_389_H variants), and SARS-CoV-2 infection (Benetti et al., 2020; Cao et al., 2020; Othman et al., 2020). The rs41303171 polymorphism, which is almost exclusive to Europeans (minor allele frequency/MAF = 1.8%), is a missense SNP causing an N_720_D replacement, which can trigger a conformational change in ACE2 affecting viral interactions (Khayat et al., 2020). A P_389_H substitution occurs in Latino American populations with a MAF of 0.015% while R_514_G, M_383_T and D_427_Y substitutions are found in African Americans with a MAF of 0.003, 0.003 and 0.01%, respectively. SARS-CoV-2 infected European people with R_708_W, R_710_C, R_710_H, or R_716_C substitutions in ACE2 usually have mild symptoms of COVID-19 as ACE2 lose the cleavage site which is the target of the TMPRSS2 protease (Hou et al., 2020). The S_19_P variant common in African populations, may also protect against COVID-19 while the K_26_R variant might predispose to severe forms of COVID-19 (Calcagnile et al., 2021). A study by Suryamohan and colleagues analyzed 290,000 samples representing more than 400 population groups and found 298 unique ACE2 variants, including the K_31_R ACE2 substitution which breaks an interaction with Q_493_ in the viral RBD and destabilizes the charge-neutralizing interaction with the virus and the E_37_K polymorphism which disrupts critical interactions with ACE2 K_353_ by removing the polar intramolecular interaction that stabilizes contact with the SARS-CoV-2 RBD (Suryamohan et al., 2021). Similarly, H_34_R was predicted to result in a loss of interface polar contact. Thus, individuals carrying these variant forms of ACE2 are predicted to be less susceptible to SARS-CoV-2 infection. Notably, the E_37_K polymorphism was found to decrease the ability of VSVDG*-SCoV-2 Beta (K_417_N/E_484_K-N_501_Y) to infect cells expressing such an ACE2 variant compared to the wild type ACE2 (Hattori et al., 2022). In contrast, these authors found that two substitutions uncommon in the global population, H_505_R (MAF = 0.001%) and Y_515_C (MAF = 0.004%), enhanced the entry of Alpha and Delta (L_452_R-T_478_K) variants and the Delta variants respectively, compared to the wild type ACE2. However, this raises the question of whether it is the Alpha variant of SARS-CoV-2 which better enters cells expressing the H_505R_ substitution in ACE2 or the H_505_R allele which favors the emergence of the SARS-CoV-2 Alpha variant by positive selection.

**Figure 5:**
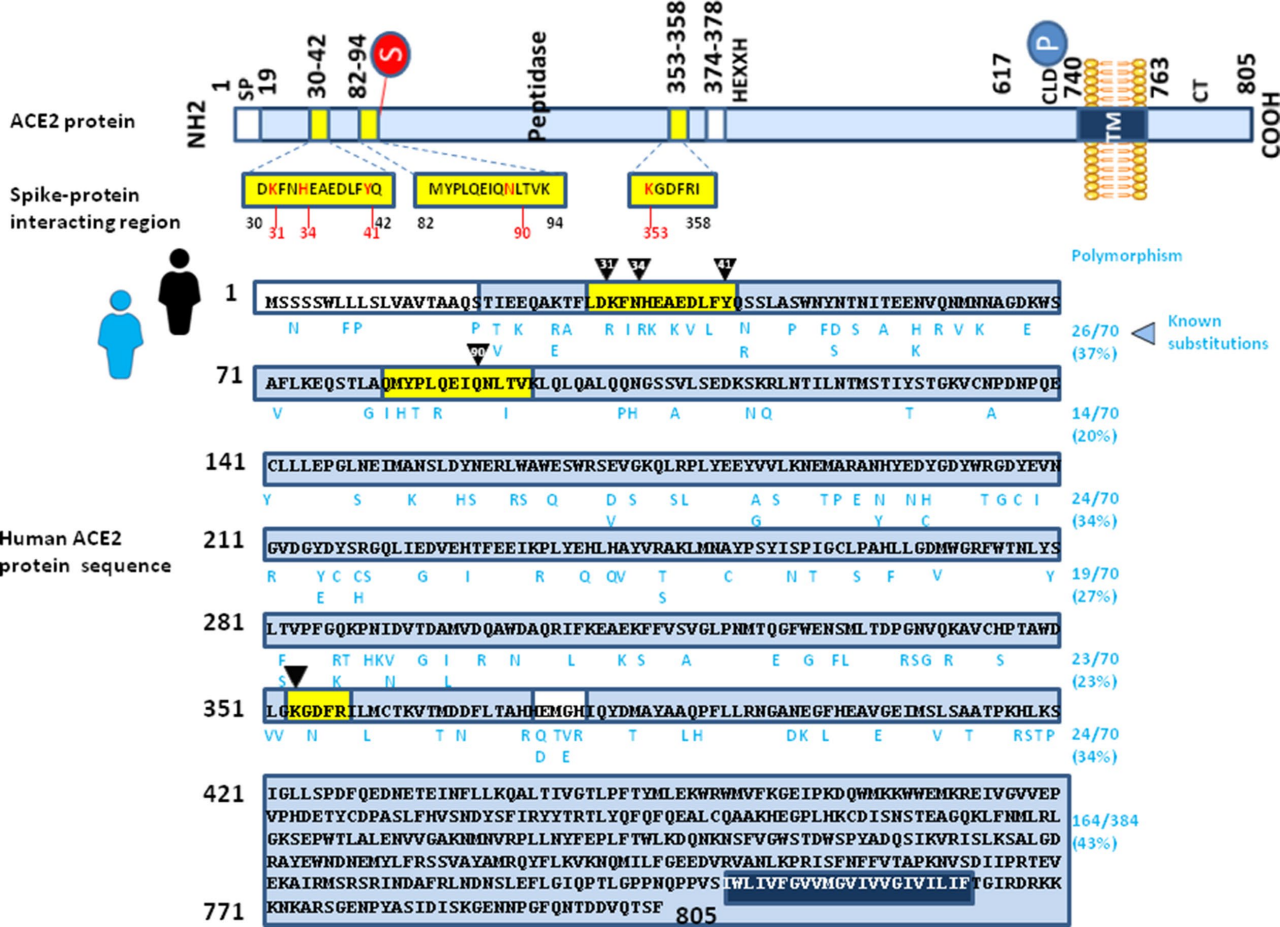
Illustration of intra-species ACE2 polymorphism. (upper panel) Schematic representation of the cell surface human ACE2 molecule and the main spike protein interacting region (see legend of Figure 2 for details). (lower panel) The figure shows the amino acids sequence of *Homo sapiens* ACE2 protein sequence (GenBank BAB40370.1). The amino acid substitutions reported in the literature for the humans ACE2 alleles are listed in a single alignment to the reference human ACE2 sequence, according to previously reported data (Suryamohan et al., 2021; Devaux and Camoin-Jau, 2022). The amino acid substitutions are shown in blue letters.

When analyzing the polymorphism of human ACE2 sequences it is noted that there is a great variability in ACE2 sequences (at least 298 positions in the human ACE2 sequence have been identified as having undergone an amino acid substitution for a protein of 805 amino acids) with substitutions which are not distributed in a homogeneous way on the entire protein. As shown in Figure 5, when we artificially cut the protein into portions of 70 amino acids, the average frequency of substitutions in the extracellular portion of the protein is between 20 and 40%. Despite the frequency of substitutions in the three main spike-protein interacting regions being not markedly different from the other regions of ACE2, there is a high frequency of substitutions affecting these regions. Although it is well known that these substitutions do not occur simultaneously on a unique human ACE2 molecule in nature, the existence of such a large number of ACE2 alleles that the virus could theoretically encounter suggests that a mechanism of ACE2-driven SARS-CoV-2 variant selection followed by a “boomerang effect” associated with human-to-human transmission could have been the source of the emergence of certain variants. In addition, the probability that SARS-CoV-2 encounters an individual carrying a minor ACE2 allele different from that carried by the individual transmitting the virus increases rapidly when it occurs in a population in which hundreds of millions of people have been infected with the virus. For example, it was reported that the K_26_R substitution in human ACE2, which is relatively frequent in European people with a MAF frequency of about 0.5% and which would correspond to a potential target population of more than two million people in the European Union, was suggested to be associated with possible increased susceptibility to COVID-19 (Calcagnile et al., 2021). The *in silico* molecular docking analysis of missense variants affecting the SARS-CoV-2 Spike RBD (including N_439_K, L_455_F, F_456_L, A_475_V, Q_493_R, Q_493_L and N_501_Y), for interaction with wild type ACE2 or K_26_R ACE2 allele was evaluated and the results indicated that several substitutions have different binding affinity for the wild type ACE2 and the K_26_R ACE2. Two ACE2 variants (E_35_K and F_72_V) possibly conferring resistance to the virus have higher allele frequencies in East Asian populations, while they have shown very low MAFs in European populations (Chen et al., 2021). The E_35_K substitution, uncommon in the global population (MAF = 0.001%) but more frequently found in East Asian population (MAF = 0.01%) seems to be neutral for the entry of the SARS-CoV-2 Alpha variant but decreases the infectivity of the Beta (K_417_N/E_484_K-N_501_Y) variant (Hattori et al., 2022). In the virus S protein, the apparent importance of K_417_ and E_484_ for interaction with ACE2 suggests the possibility that K_417_N and E_484_K substitutions should negatively affect binding to the ACE2 E_35_K mutant.

## 9. Three-dimensional study of SARS-CoV-2 S protein binding to rare ACE2 alleles suggests that an ACE2 -driven “boomerang effect” also applies during human-to-human transmission of SARS-CoV-2

Several human ACE2 variants (I_21_V, G_23_K, K_26_R, N_64_K, T_92_I, Q_102_P, D_206_G, G_211_R, R_219_C, E_329_G, H_378_R, V_447_F, A_501_T and N_720_D) which are thought to increase susceptibility to SARS-CoV-2 have higher allele frequencies in European populations than in East Asian populations (Chen et al., 2021). Thus, the effect of ACE2 substitutions on the entry of SARS-CoV-2 variants might differ among SARS-CoV-2 variants and could be seen as a positive selection factor affecting the spike sequence.

Notably, the early N_501_Y lineage (501Y variant 1) co-circulated with the N_501_ lineage between early September and mid-November 2020 in Wales, were it never became dominant (the 501Y variant 1 never exceeded 2% among the sequenced samples), whereas a later N_501_Y lineage (501Y variant 2, also named UK variant B1.1.7), harboring 14 non synonymous mutations and three deletions across its viral genome, emerged in October/September 2020 in England and rapidly became dominant (the 501Y variant 2 represented 49.7% among the sequenced samples in November) (Leung et al., 2021). The N_501_Y substitution found in the B1.1.7 variant was considered to be a critical determinant of enhanced infection of this highly transmissible variant (Leung et al., 2021; Liu et al., 2022). One recent study suggested that the best explanation for the origin of the B1.1.7 variant would be a chronically infected individual rather than a non-human animal population (Hill et al., 2022). Thus, the N_501_Y UK variant B1.1.7 was a good candidate for testing our hypothesis of ACE2-driven SARS-CoV-2 variant selection during intraspecies transmission of the virus among European people expressing rare ACE2 alleles.

**Figure 6:**
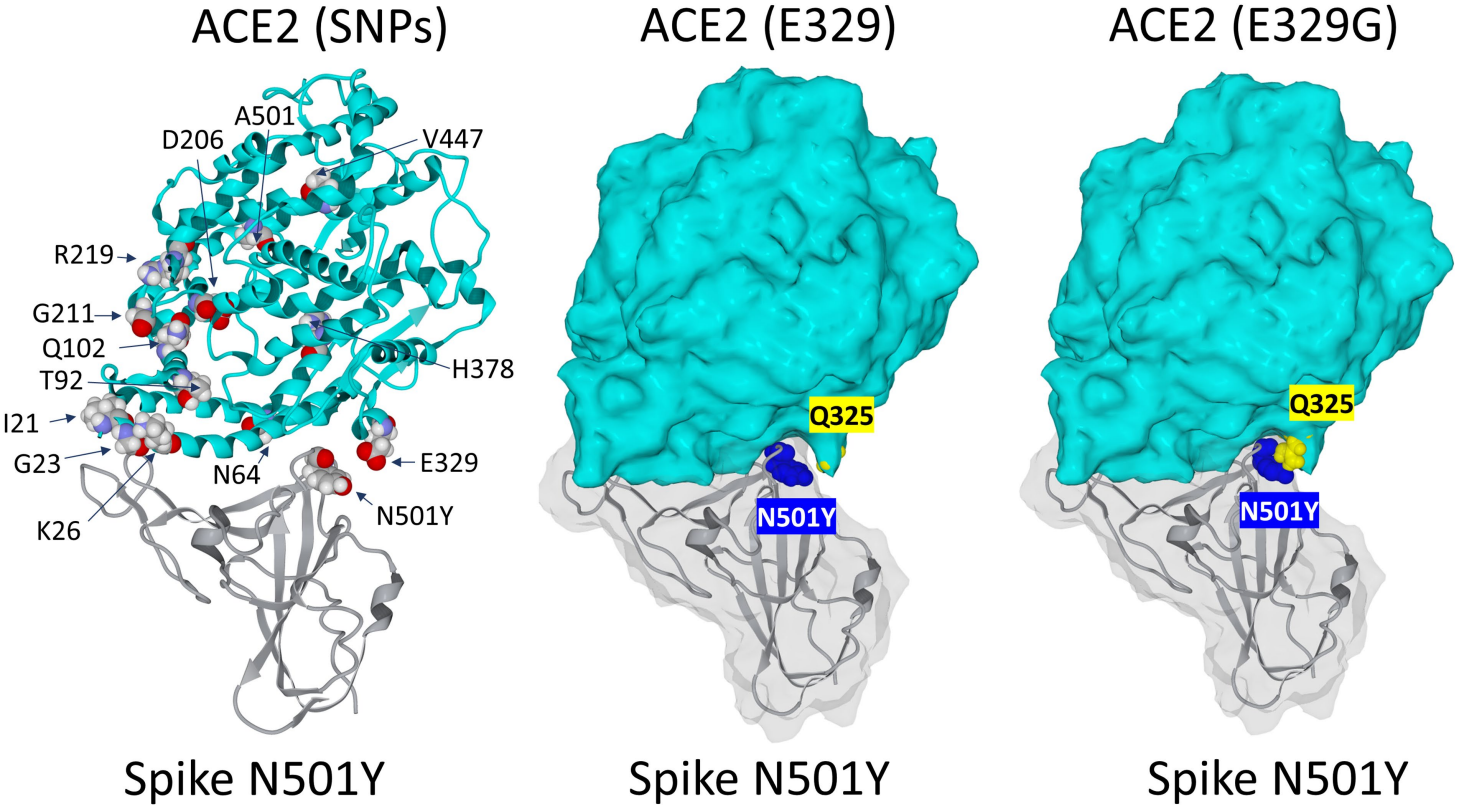
Impact of the rs143936283 single nucleotide polymorphism (SNP) leading to E_329_G substitution in ACE2 on the interaction with the SARS-CoV-2 N501Y Spike protein mutant. Left panel: localization of major substitutions in the 3D structure of the ACE2 receptor in complex with the N_501_Y receptor binding domain (RBD) of the SARS-CoV-2 spike protein. Middle panel: visualization of Q_325_ (yellow atomic spheres) relatively to N_501_ (blue atomic spheres) on the complex represented in surface rendition. Note that there is no direct contact between N_501_Y and E_325_ and that the surface of ACE2 does not fit well with N_501_Y in this part of the complex. Right panel: the E_329_G substitution of ACE2 (yellow atomic spheres) allows Q_325_ to come closer to N_501_ (blue atomic spheres). In this case, the fit between ACE2 and the mutated spike protein has been significantly improved.

In order to assess the impact of ACE2 SNPs on the affinity of this receptor for the SARS-CoV-2 spike protein with the N_501_Y mutation, we located the main amino acid substitutions in the 3D structure of ACE2 (Figure 6). It emerged from this analysis that no position seems to have a direct effect on the interaction with the mutated spike protein. This is because most substitutions are distant from tyrosine-501. In fact, the closest substitution to N_501_Y is the amino acid residue E_329_. However, this residue does not interact directly with N_501_Y. We then looked for a possible effect of the E_329_G mutation in the polymorphisms of ACE2. Our molecular docking analysis revealed an indirect mechanism by which the polymorphism at position 329 can influence the binding of SRAS-CoV-2 spike protein bearing the N_501_Y mutation. This indirect effect involves the side chain of glutamine Q_325_ whose position differs depending on the polymorphism of E_329_. The structural models established in the two cases (E_329_ and E_329_G) clearly show that the E_329_G polymorphism is much more favorable to an interaction with the mutated spike protein N_501_Y than E_329_ (Figure 6). Thus, we can see a physical contact being established between N_501_Y and Q_325_ for ACE2 with E_329_G, whereas this contact does not appear for ACE2 with E_329_.

**Figure 7:**
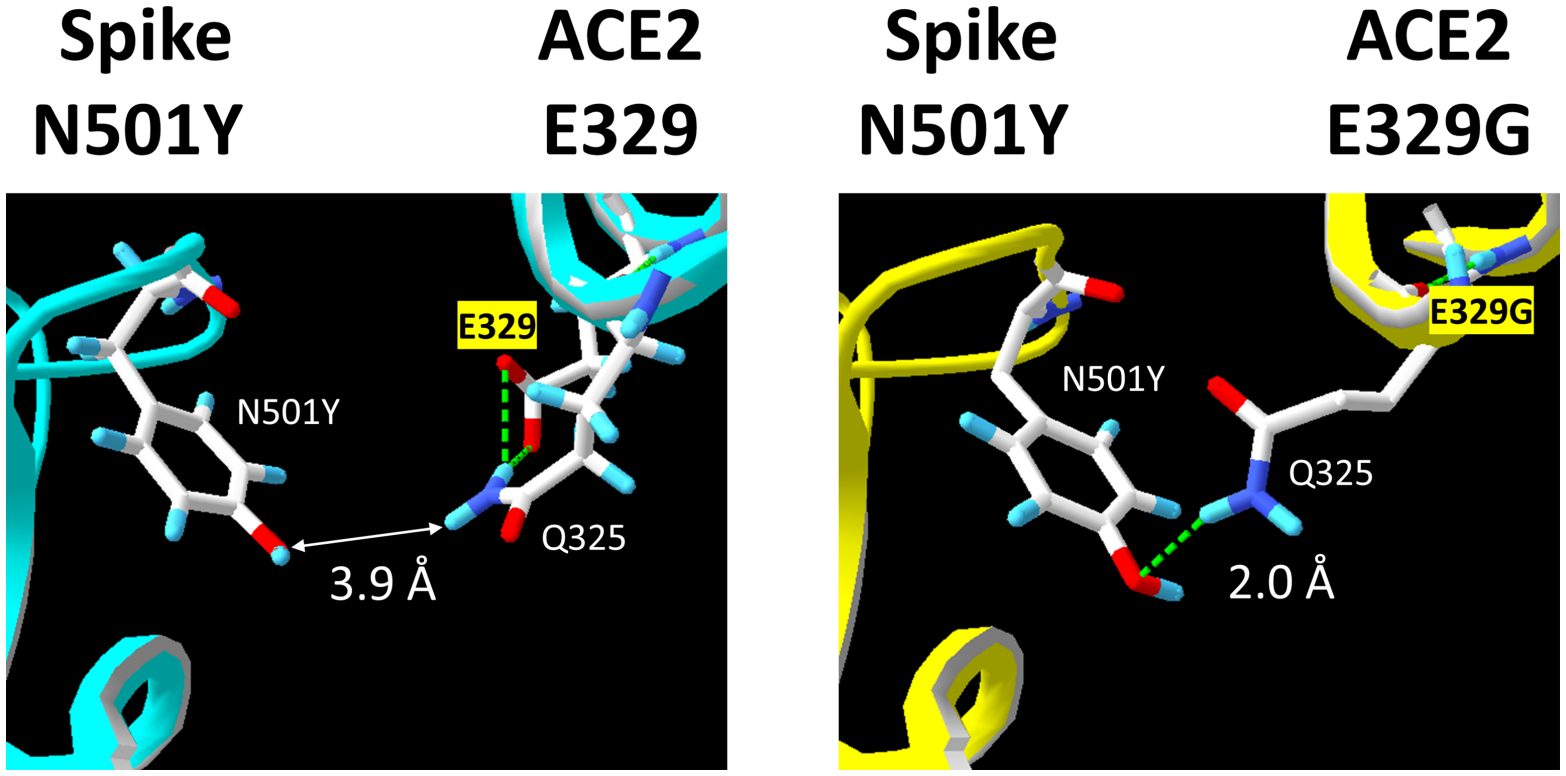
Molecular mechanism explaining how the polymorphism at position 325 of ACE2 controls the interaction with the N_501_Y spike protein. Left panel: in the case of the E_329_ ACE2, two H bonds (green dashed lines) maintain the side chain of Q_325_ close to the ACE2 surface, preventing any contact with N_501_Y. Right panel: when E_329_ is replaced by E_329_G, these H bonds are abolished, so that the side chain of Q_325_ can now physically interact with N_501_Y through a stabilizing H bond (green dashed line).

Our modeling studies allowed us to decipher the molecular mechanism explaining why E_329_G is particularly favorable to an interaction with N_501_Y. Glutamic acid at position 329 (E_329_) attracts the side chain of Q_325_ to itself and stabilizes it to the surface of ACE2 by forming two H bonds (Figure 7). Under these conditions, the side chain of Q_325_ is not available to interact with N_501_. However, if ACE2 presents a glycine instead of this glutamic acid (E_329_G), these two H bonds are abolished and the side chain of Q_325_ is then free to come into close contact with N_501_Y. An H bond stabilizes the complex and the two residues being then separated by only 2.0 Å, against 3.9 Å in the case of E_329_. This reorganization leads to a 5.9 fold multiplication of the interaction energy of the S protein-ACE2 complex at the level of the 325–330 region of ACE2. It is therefore clear that ACE2 displaying the E_329_G mutation is significantly more favorable to selecting a virus with a spike protein presenting the N_501_Y mutation than its E_329_ counterpart.

## 10. Discussion

In this study we report evidence that adaptation to the ACE2 polymorphism is a major determinant of the so-called “multiple waves” of the SARS-CoV-2 pandemic through lineage replacement. It is now well established that the inter-species transmission of the virus can lead to finding SARS-CoV-2 carrying mutations that have been selected specifically for viral fitness to ACE2 orthologs (e.g., selection of minor mutants in quasispecies), such as the specific Y_453_F substitution aimed to improve the viral spike binding to the mink ACE2 or by associated compensatory mutations. When we compare the risk to humans of an adaptation of SARS-CoV-2 of human origin to the mink ACE2 and the hamster ACE2, we note that the risk represented by adaptation to mink ACE2 is low while it becomes relatively high in the case of adaptation to hamster ACE2.

Other inter-species transmission events are likely to be at the origin of lineage replacement in humans. As indicated earlier in this hypothesis paper, the B.1.1.529/Omicron lineage (BA.1) and its subvariants have surpassed all other SARS-CoV-2 lineages and are the ones that maintain a very high level of infection in the human population today. The spike protein of this lineage contained 45 point mutations compared with the B1.1 lineage, including seven changes in the N-terminal domain (NTD), a scale of mutations never before observed with other SARS-CoV-2 lineages. The high number of substitutions found at the interface of ACE2 indicates a specific genetic drift to better fit the ACE2 of its main animal host, likely to be mouse ACE2 (Wei et al., 2021). One recent article indicated that the XBB and XBB.1 subvariants of SARS-CoV-2 Omicron BA.2 and the BQ.1 and BQ.1.1 subvariants of BA.5 are now growing rapidly, probably due to mutations in the S gene enabling the virus to evade the host immune responses (Wang et al., 2023). Wang and colleagues reported that the capacity of sera from vaccinated persons to neutralize BQ.1, BQ.1.1, XBB and XBB.1 (recombination variants) was markedly impaired, including sera from individuals boosted with a bivalent WA1/BA.5 mRNA vaccine. The titers against the BQ and XBB subvariants were 13- to 81-fold and 66- to 155-fold lower, respectively, well above what has been observed to date. Monoclonal antibodies capable of neutralizing the original Omicron variant are in fact largely inactive against these newer subvariants. However, these were found to have similar ACE2 binding affinities compared to their predecessors. Recently, Yue and colleagues reported that novel XBB1.5 variant had similar spike binding affinity with BA2.75, but had higher binding affinity than XBB.1 and BQ.1.1 (Yue et al., 2023). These results indicate that the BQ and XBB (recombinant) subvariants pose serious threats to current COVID-19 vaccines and may have acquired a dominant position in the population due to their neutralizing antibody evasion advantage.

Of course, all the different lineages of SARS-CoV-2 that have circulated in humans since the beginning of the pandemic are not the result of an inter-species transmission and the question arises as to whether these variants are the products of stochastic events or whether they have been determined by a polymorphism of ACE2 in humans. Given the apparent absence of selective immune system pressure during the pre-Omicron and pre-vaccine pandemic period, mutations N_501_Y (Alpha, Beta, Gamma), P_681_H (Alpha, Delta), K_417_N (Beta and Gamma), and E_484_A (Beta and Gamma) emerged, thus increasing the stability of the trimeric Spike and potentially improving its interaction with ACE2 (Martin et al., 2021; de Lima et al., 2022). These observations suggest a model of convergent adaptive evolution. Several Omicron sublineages show evidence of mutations in their RBD, despite recent mutation. For example, the BA.2.3 that already harbored the E_484_A inherited from the BA.2, further mutated into A_484_R in the child BA.2.3.20, which caused a high increase in ACE2 affinity to which K_444_R, L_452_M, and N_460_K also contributed. There are many other examples into the Omicron lineage of missense mutations leading to increased affinity for ACE2 (Focosi et al., 2023). Notably, even recombinant viruses could be subject to ACE2 selection. For example, our institute reported the identification of the Delta 21J_AY.4-Omicron 21 K/BA.1 “Deltamicron” recombinant, composed of the near full-length spike gene of an Omicron 21 K/BA.1 variant in a Delta 21 J/AY.4 lineage backbone and the structural analysis of the recombinant spike suggested its hybrid content could optimize viral binding to the host cell membrane and increase the electrostatic surface potential of the RBD. This in turn may facilitate the interaction with the electronegative interface of the ACE2 cellular receptor (Colson et al., 2022b). The results of inter-species transmission strongly suggest that what is observed with the viral dynamics over time favor a similar ACE2-driven selection of variant followed by “boomerang effect” at the level of human-to-human transmission during the pandemic. We used *in silico* modeling of SARS-CoV-2 spike/human ACE2 interaction to test this hypothesis.

The N_501_Y substitution in the SARS-CoV-2 spike is required for the adaptation of a “human” SARS-CoV-2 to a murine ACE2 (Wei et al., 2021). However, at least in Europe, the N_501_Y substitution in the SARS-CoV-2 spike likely emerged during virus selection in humans without any previously known inter-species transmission. A first N_501_Y lineage (501Y variant 1) emerged in September 2020 in Wales, followed another N_501_Y lineage (501Y variant 2, also named UK variant B1.1.7) in October 2020 in England which rapidly became dominant (Leung et al., 2021; Hill et al., 2022; Liu et al., 2022). Among the different rare ACE2 alleles identified by geneticists, we focused our attention to the rs143936283 single nucleotide polymorphism leading to E_329_G substitution in ACE2. The impact of this substitution on interaction with the SARS-CoV-2 spike was previously debated (Hussain et al., 2020; Ragia and Manolopoulos., 2020; Chen et al., 2021). According to Hussain and colleagues, the rs143936283 (E_329_G) ACE2 variant showed less charged-charged, charged-polar, charged-apolar, polar-apolar, and apolar-apolar interactions with the SARS-CoV-2 spike protein, suggesting that expression of the ACE2 allele E_329_G variant in humans may confer some level of resistance against the attachment of SARS-CoV-2 to its receptor molecule. Further investigation of inter-residual interaction indicated that for the consensus human ACE2, the E_35_ amino acid of ACE2 interacts with the SARS-CoV-2 spike protein Q_493_, while this interaction is absent for the ACE2 allele E_329_G variant. A similar observation was made for Q_42_ of ACE2 which usually interacts with the SARS-CoV-2 spike protein Y_449_, suggesting that ability of E_35_ and Q_42_ to interact with the SARS-CoV-2 spike protein could be affected by their spatial positioning in the ACE2 protein, which may be affected by the change in the intramolecular interaction and/or electrostatic potential due to the substitution in the flanking residues with respect to the three-dimensional conformation of ACE2. In addition, the ACE2 allele E_329_G variant, which was predicted to have lower binding affinity for the SARS-CoV-2 spike protein, lacked the hydrogen bond interaction between the K_353_ of the ACE2 and the G_502_ of the SARS-CoV-2 spike protein in the complex, suggesting a possible intrinsic resistance against the SARS-CoV-2 infection. This relatively intrinsic resistance to SARS-CoV-2 may have been a good reason for selecting a virus better adapted to a higher affinity interaction for this ACE2 allele E_329_G variant. This SNP is uncommon in the global population (and is absent in Asian people) but is sometimes found in the European population (MAF = 0.02%) (https://www.ncbi.nlm.nih.gov/snp/rs143936283#frequency_tab; accessed March 26, 2023). This allele frequency is to be compared to the more than 2.28 million cases of SARS-CoV-2 infections in England during the year 2020 (https://www.gov.uk/government/organisations/uk-health-security-agency; accessed on March 26, 2023), which raises the possibility that a SARS-CoV-2 quasispecies with a dominant N_501_ spike and undetectable N_501_Y variant chronically infected an immuno-deficient individual carrying the rs143936283 allele of ACE2. Thus, the N_501_Y UK variant B1.1.7 was a good candidate for testing our hypothesis of ACE2-driven SARS-CoV-2 variant selection during intraspecies transmission of the virus among European people expressing rare ACE2 alleles. Our molecular docking analysis revealed a mechanism by which the E_329_G substitution in ACE2 can influence the binding of the SARS-CoV-2 spike protein bearing the N_501_Y mutation and established that the E_329_G polymorphism is much more favorable to an interaction with the mutated spike protein N_501_Y than E_329_. Thus, we demonstrated physical contact between N_501_Y and Q_325_ for an uncommon ACE2 allele with a E_329_G substitution, whereas this contact does not appear for the most common ACE2 alleles with E_329_. Very recently, we analyzed mutations in a large set of 61,397 SARS-CoV-2 genomes sequenced in our institute for COVID genomic surveillance during the entire period of the pandemic. A total of 22,225 nucleotide mutations were identified, 220 (1.0%) being classified as “hyperfertile” (found at very high frequency) (Colson et al., 2023).Within the Spike gene, 61,214 sequences had the D_614_G substitution and 25,345 sequences had the N_501_Y substitution, confirming the importance of the “hyperfertile” N_501_Y mutation (which ranks 5th in terms of the most frequently encountered mutations in the viral S protein) in the evolution of SARS-CoV-2 in humans.

Of course, we do not have formal proof that the emergence of the Alpha B1.1.7 variant occurred through selection by the rs143936283 (E_329_G) rare ACE2 allele, but it suggests that such a mode of selection is entirely possible. Although this is only one piece of the puzzle [other positive selection mechanisms such as immune response do exist (Harvey et al., 2021; Carabelli et al., 2023; Snouwaert et al., 2023)], taken together our data indicate that the ACE2-driven selective sweep and “boomerang effect” are very important parameters which contribute to the evolution of SARS-CoV-2 lineages over time. This model is perfectly in line with the recent results of Yue and colleagues (Yue et al., 2023) which indicate that enhanced transmissibility of XBB.1.5 is contributed by both strong ACE2 binding and antibody evasion. It is also very likely that this model could be extrapolated to other viruses and their respective cellular receptors.

## Data availability statement

Publicly available datasets were analyzed in this study. This data can be found here: We have used SARS-CoV-2 protein sequences freely available from GISAID and ACE2 protein sequences freely available through GenBank. All the sequences and methods have been previously reported in our published papers (Devaux et al., 2021a,b; Fantini et al., 2021a,b; Frutos et al., 2022).

## Author contributions

All authors listed have made a substantial, direct, and intellectual contribution to the work and approved it for publication.

## Funding

This work was supported by the French Government under the “Investissements d’avenir” (Investments for the Future) program managed by the Agence Nationale de la Recherche (ANR, FR: National Agency for Research), (reference: Méditerranée Infection 10-IAHU-03) and an annual budget allocation from the Aix-Marseille University and the “Institut de Recherche pour le Développement” (IRD) to the Microbes Evolution Phylogeny and Infection (MEPHI) laboratory.

## Conflict of interest

CD declares a link of interest with the Sanofi and Merck pharmaceutical companies. JF declares that the research was conducted in the absence of any commercial of financial relationships that could be construed as a potential conflict of interest.

## Publisher’s note

All claims expressed in this article are solely those of the authors and do not necessarily represent those of their affiliated organizations, or those of the publisher, the editors and the reviewers. Any product that may be evaluated in this article, or claim that may be made by its manufacturer, is not guaranteed or endorsed by the publisher.

## Footnotes

^1^ https://vis.csh.ac.at/sars-ani/
