# Supplementary material for: ACE2 receptor polymorphism in humans and animals increases the risk of the emergence of SARS-CoV-2 variants during repeated intra- and inter-species host-switching of the virus
Source: Front Microbiol. 2023 Jul 13;14:1199561. doi: 10.3389/fmicb.2023.1199561 (PMC10373931; doi:10.3389/fmicb.2023.1199561)
Supplement: Supplementary file 1 [file Data_Sheet_1.PDF]

Supplementary Material

ACE2 receptor polymorphism in humans and animals increases the risk of the emergence of SARS-CoV-2 variants during repeated intra- and inter-species host-switching of the virus

Christian A. Devaux*, **Jacques Fantini**

*** Correspondence:** Corresponding Author: christian.devaux@mediterranee-infection.com

1 Supplementary Data

SARS-CoV-2 circulation in animal species (adapted from a Vetmeduni report).

2 Supplementary Figure S1

3 Supplementary Figure S2

Supplementary Figure S1. SARS-CoV-2 circulation in animal species (adapted from a Vetmeduni report). According to the reference site Vetmeduni/Complexity Science Hub, Vienna reference site (https://vis.csh.ac.at/sars-ani/) which reported an overview of SARS-CoV-2 events in animals between January 2020 and December 2022. 616 outbreaks of SARS-CoV-2 had been reported in 31 animal species with a 2.8% fatality rate occurring in 39 countries.

Supplementary Figure S2

Clustal Omega multiple sequence alignment (EMBL-EBI bioinformatic tool; Copyright EMBL 2020) was used to compare the protein sequences of Homo sapiens (Hum) ACE2 (GenBank: BAB40370.1) and deer (Cervus elaphus; CervusE) ACE2 (NCBI databank: XP_043752042.1) The amino acids that differ between deer and human are shown in yellow. Amino acid sequences important for the viral S protein binding to ACE2 are indicated by an arrow.
