# Supplementary figures and images for: ACE2 receptor polymorphism in humans and animals increases the risk of the emergence of SARS-CoV-2 variants during repeated intra- and inter-species host-switching of the virus
Source: Front Microbiol. 2023 Jul 13;14:1199561. doi: 10.3389/fmicb.2023.1199561 (PMC10373931; doi:10.3389/fmicb.2023.1199561)

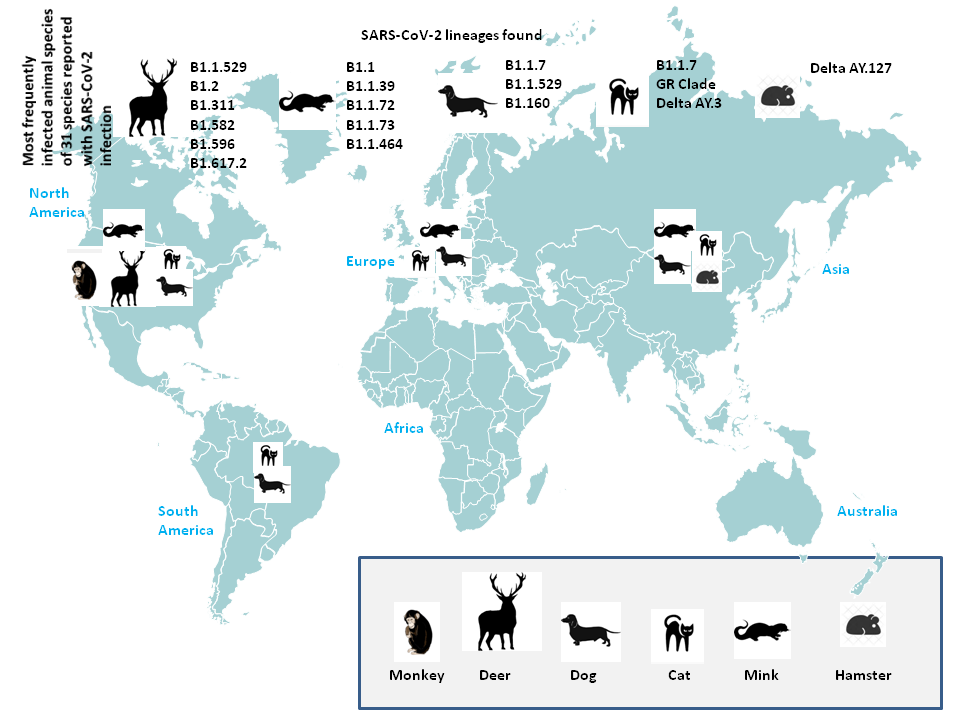

Supplement: Supplementary file 2 [file Image_1.TIF]

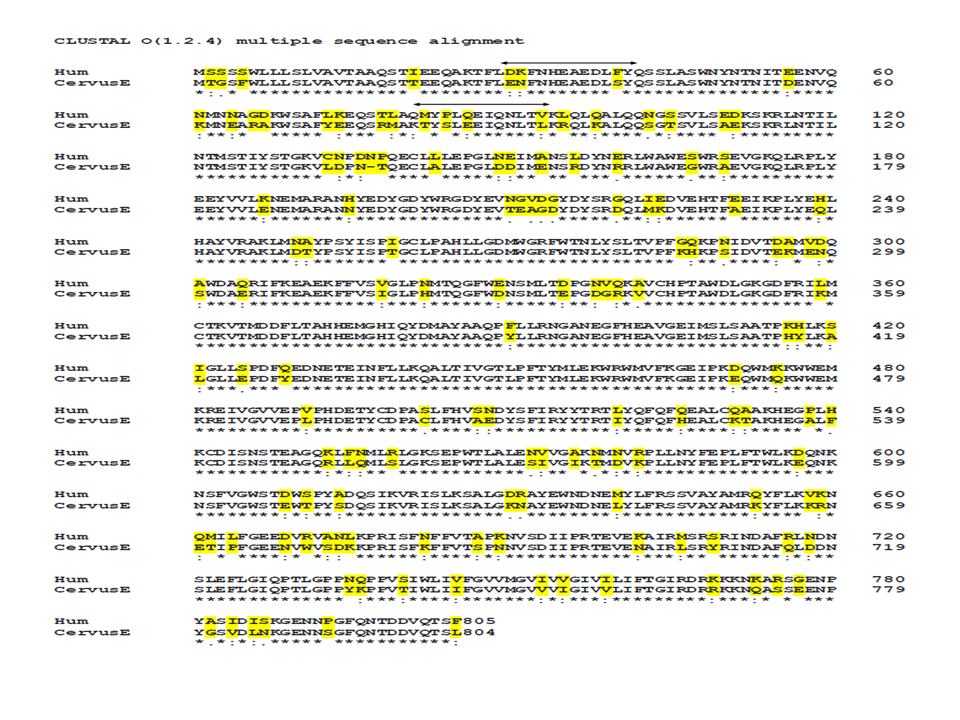

Supplement: Supplementary file 3 [file Image_2.TIF]
